# Effect of F16-Betulin Conjugate on Mitochondrial Membranes and Its Role in Cell Death Initiation

**DOI:** 10.3390/membranes11050352

**Published:** 2021-05-10

**Authors:** Mikhail V. Dubinin, Alena A. Semenova, Darya A. Nedopekina, Eldar V. Davletshin, Anna Yu. Spivak, Konstantin N. Belosludtsev

**Affiliations:** 1 Department of Biochemistry, Cell Biology and Microbiology, Mari State University, pl. Lenina 1, 424001 Yoshkar-Ola, Russia; sem_al.ru@mail.ru (A.A.S.); bekonik@gmail.com (K.N.B.); 2 Institute of Petrochemistry and Catalysis, Russian Academy of Sciences, Prospekt Oktyabrya 141, 450075 Ufa, Russia; rawbe2007@mail.ru (D.A.N.); eldarik1996@mail.ru (E.V.D.); spivak.ink@gmail.com (A.Y.S.); 3 Institute of Theoretical and Experimental Biophysics, Russian Academy of Sciences, Institutskaya 3, 142290 Pushchino, Russia; 4 Prokhorov General Physics Institute, Russian Academy of Sciences, Vavilova 38, 119991 Moscow, Russia

**Keywords:** F16, betulin, thymocytes, mitochondria, oxidative phosphorylation, ROS

## Abstract

This work demonstrates the effects of a newly synthesized conjugate of the plant triterpenoid betulin and the penetrating cation F16 used for mitochondrial targeting. The resulting F16-betulin conjugate revealed a mitochondria-targeted effect, decreasing the mitochondrial potential and inducing superoxide overproduction in rat thymocytes *in vitro*. It has been suggested that this may cause the cytotoxic effect of the conjugate, which significantly exceeds the effectiveness of its precursors, betulin and F16. Using isolated rat liver mitochondria, we found that the F16-betulin conjugate has a surface-active effect on mitochondrial membranes, causing organelle aggregation. This effect of the derivative resulted in a dose-dependent decrease in mitochondrial transmembrane potential, as well as suppression of respiration and oxidative phosphorylation, especially in the case of nicotinamide adenine dinucleotide (NAD)-fueled organelles. In addition, the F16-betulin conjugate caused an increase in H_2_O_2_ generation by mitochondria fueled with glutamate and malate. These effects of the derivative can presumably be due to the powerful suppression of the redox activity of complex I of the mitochondrial electron transport chain. The paper discusses how the mitochondria-targeted effects of the F16-betulin conjugate may be related to its cytotoxic effects.

## 1. Introduction

Triterpenoids, widespread in the plant kingdom, are well-known modifiers of biological and artificial membranes [1]. This property of triterpenes is widely used in the synthesis of new therapeutic agents that, among other things, have a targeted effect. In particular, a popular trend is the creation of synthetic hybrids based on triterpenes and mitochondria-targeted delocalized cations, which can be used to modulate the activity of membrane proteins and enzymes of these organelles [2,3,4,5,6]. It is well known that mitochondria not only support the functioning of healthy cells, but are also involved in neoplasia and differentiation of cancer cells [7,8,9]. Due to the fact that the mitochondria of many solid tumor cells have a high transmembrane potential, which exceeds the potential of normal cells [10], there is a possibility for the selective accumulation of lipophilic molecular hybrids based on triterpenes and penetrating delocalized cations in the tumor cell mitochondria and modulation of their activity.

**Figure 1:**
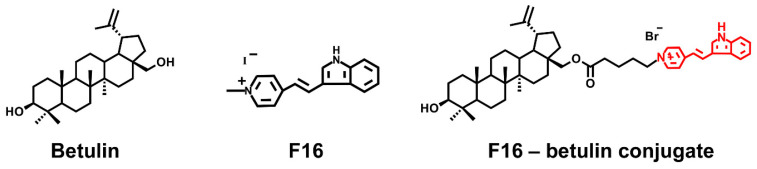
Structure of betulin, F16 ([E-4-(1H-indol-3-ylvinyl)-N-methylpyridinium iodide]) and of F16-betulin conjugate.

The main chemical agents successfully used to create hybrid molecules that can be targeted to mitochondria include delocalized lipophilic cationic compounds (DLCs) such as rhodamine 123, F16, MKT-077, and lipophilic cationic dequalinium and triphenylphosphonium salts [11,12,13,14,15,16,17,18]. Available natural pentacyclic triterpenoids, represented by betulin, as well as betulinic, glycyrrhetic, oleanolic and ursolic acids, may be used as promising scaffolds to create functionally active conjugates showing pro- or antioxidant properties. The antitumor effect of native triterpenes has been well studied *in vitro* on various lines of tumor cells and is due to the overproduction of ROS particles, leading to cell death and elimination of cancer cells [19,20,21]. However, the poor water solubility of these triterpenes requires the use of high concentrations to accumulate in mitochondria and achieve the desired therapeutic effect, and this may cause numerous side effects that limit their use in clinical medicine. At the same time, triphenylphosphonium (or Rhodamine B)-linked triterpenoid derivatives show a much higher antitumor activity, inducing mitochondria-mediated apoptosis of tumor cells [2,3,4,5,6]. One of the promising penetrating cations showing mitochondrial targeting is the lipophilic delocalized F16 cation exhibiting anticancer activity in the free form [22], which, however, is manifoldly enhanced upon hybridization with triterpenes. Indeed, we recently found that conjugates of F16 and pharmacologically active betulinic acid have a targeted effect on mitochondria, inducing ROS overproduction and permebilization of organelle membranes [6]. In addition, we have synthesized the conjugate of native betulin and F16 (Figure 1) and demonstrated that submicromolar concentrations of this agent had a powerful antitumor effect, significantly exceeding the activity of the parent F16 cation (>800 times) [23].

An important task in the development of new drugs is to determine the therapeutic window and assess their effect on healthy cells. Therefore, in this work, we evaluated the effect of the new F16-betulin conjugate on the thymocytes of laboratory rats, and also studied the role of mitochondria in the cytotoxic action of this agent. We also estimated the membranotropic effect of the new derivative on mitochondria isolated from rat liver and assessed conjugate-induced changes in key parameters reflecting mitochondrial function: (1) intensity of respiration and oxidative phosphorylation; (2) activity of electron transport chain complexes; (3) reactive oxygen species (ROS) generation intensity. The results obtained suggest that the cytotoxic effect of the new conjugate is due to its selective accumulation in mitochondria causing mitochondrial dysfunction and ROS overproduction. The F16-betulin conjugate has been shown to have a significant effect on the surface properties of mitochondrial membranes, which can lead to a change in the activity of complexes of the mitochondrial electron transport chain and, in particular, a powerful inhibition of complex I most likely leading to ROS overproduction.

## 2. Materials and Methods

### 2.1. Synthesis of F16-Betulin Conjugate

F16-betulin conjugate was synthesized from commercially available betulin, which was transformed into C-28 bromoalkyl ether by reaction with 5-bromovaleric acid in dry methylene chloride using dicyclohexylcarbodiimide and 4-dimethylaminopyridine according to the method [24]. The resulting bromoalkyl ether was reacted with (E)-4-(1H-indol-3-ylvinyl)pyridine by refluxing in CH_3_CN or in DMF at 85 °C in an argon atmosphere for 12 h, followed by purification of the conjugate by column chromatography on SiO_2_. The neutral precursor F16-(E)-4-(1H-indol-3-ylvinyl)-pyridine was obtained by reacting gramine with pyridine-4-carbaldehyde using tri-*n*-butylphosphine in CH_3_CN at 81 °C under argon atmosphere for 24 h [25].

The structure of product was confirmed by 1D (^1^H, ^13^C, APT), 2D homonuclear correlation spectroscopy (COSY), nuclear Overhauser effect spectroscopy (NOESY) and heteronuclear single quantum coherence (HSQC), heteronuclear multiple bond correlation (HMBC) NMR experiments. The nuclear chemical shifts for the terpene core and for (E)-4-(1H-indol-3-ylvinyl)pyridine were determined by comparison with published data [3,4,23,26,27]. In the ^1^H NMR spectra, the presence of (E)-4-(1H-indol-3-ylvinyl)pyridinium moiety is evidenced by two characteristic doublets for the pyridine ring at 8.02 and 8.58 ppm with J = 6.0 Hz, two vinyl group doublets at 7.10 and 8.09 ppm with J = 16.0 Hz, and three characteristic multiplets for the indole moiety at 7.22–7.29, 7.48–7.88 and 8.07–8.09 ppm. The ^13^C NMR spectra show signals for the (E)-4-(1H-indol-3-ylvinyl)pyridinium carbons in the 113.6–157.4 ppm range.

### 2.2. Isolation of Rat Thymocytes and Flow Cytometry

Thymocytes were isolated from 3 thymuses of Wistar rats (animal’s weight was 110–140 g) in accordance with a known method [28] (approved by the Mari State University Ethics Committee, decision number: 11/2020 on 10. November 2020). Medium containing 145 mM NaCl, 5.6 mM KCl, 8 mM Mops (pH 7.4) and 10 mM glucose was used to isolate, wash, suspend and incubate the cells. In control samples, the cell survival rate was at least 78–83%. All cell experiments were carried out for 3 h; in this case, the cells retained similar viability, ROS profile and mitochondrial potential, assessed by flow cytometry using Muse Cell Analyzer (Merck Millipore, Burlington, MA, USA).

The cell viability and count after 15 min treatment with the test compounds was studied by flow cytometry using Muse Count & Viability Kit (MCH100102). Mitochondrial potential was assessed by using Muse MitoPotential Kit (Merck Millipore, Burlington, MA, USA) in order to determine the percentages of cells exhibiting a change in mitochondrial polarization. The quantitative measurement of cellular populations undergoing oxidative stress was performed using the Muse Oxidative Stress Kit (MCH100111) (Merck Millipore, Burlington, MA, USA) based on the superoxide-sensitive dihydroethidium dye. All assays were done strictly under the manufacturer’s protocol.

### 2.3. Isolation of Rat Liver Mitochondria

Mitochondria were isolated from the liver of 110–140 g male Wistar rats using a convenient technique of differential centrifugation [29]. The isolation medium contained 250 mM sucrose, 1 mM EGTA and 5 mM Hepes/KOH buffer (pH 7.4). The mitochondrial protein concentration was determined by the biuret method with bovine serum albumin (BSA) used as standard.

### 2.4. Optical Microscopy Imaging of Mitochondrial Samples

Optical microscopy of mitochondria was performed in the medium composed of 210 mM mannitol, 70 mM sucrose, 1 mM KH_2_PO_4_, 5 mM succinic acid, 2 μM rotenone, 10 μM EGTA and 10 mM Hepes/KOH buffer, pH 7.4. After incubation for 10 min with studied agent, 10 µL of mitochondrial suspension was placed on the glass slide and covered with the cover glass to capture the images using the EVOS M5000 Imaging System (Thermo Fisher Scientific, Waltham, MA, USA).

### 2.5. Monitoring Optical Density of Mitochondrial Suspension

The change in optical density of the mitochondrial suspension was estimated at 540 nm using a plate reader Multiskan GO (Thermo Fisher Scientific, Waltham, MA, USA). Rat liver mitochondria (0.5 mg/mL) were incubated in the medium containing 210 mM mannitol, 70 mM sucrose, 1 mM KH_2_PO_4_, 10 μM EGTA and 10 mM Hepes/KOH buffer, pH 7.4. 2.5 mM glutamate *plus* 2.5 mM malate or 5 mM succinate were used as respiratory substrates. In the latter case, the incubation medium was supplemented with 1 μM rotenone.

### 2.6. Determination of Mitochondrial Respiration and Oxidative Phosphorylation

The rate of oxygen consumption was measured polarographically with a Clark-type gold electrode (O2k, OROBOROS Instruments, Innsbruck, Austria) at 25 °C under continuous stirring [30]. Rat liver mitochondria (0.5 mg/mL) were incubated in the medium containing 200 mM sucrose, 20 mM KCl, 0.5 mM EGTA, 5 mM KH_2_PO_4_ and 10 mM Hepes/KOH, pH 7.4. Next, 2.5 mM glutamate *plus* 2.5 mM malate or 5 mM succinate were used as respiratory substrates. In the latter case, the incubation medium was supplemented with 1 μM rotenone. Estimated were the mitochondrial respiration in resting state (i.e., basal mitochondrial respiration in the presence of exogenous substrates or state 2), in state 3 (exogenous substrates plus ADP), in state 4 (after ADP exhaustion), uncoupled state 3U_DNP_ in the presence of an uncoupler (2,4-dinitrophenol) [30]. The rates of substrate oxidation were expressed as nmol O_2_/min/mg mitochondrial protein. Respiratory control ratio (RC = state 3/state 4).

### 2.7. Measuring Activity of Complexes of the Mitochondrial Electron Transport Chain (ETC)

The effect of derivative on the activity of mitochondrial electron transport chain complexes (I, II, III, IV) was estimated using specific redox reactions according to the protocol using a Multiskan GO plate reader (Thermo Fisher Scientific, Waltham, MA, USA) [31]. The concentration of mitochondrial protein was about 50 µg/mL.

### 2.8. Monitoring of Mitochondrial Membrane Potential

The mitochondrial Δψ was evaluated with the fluorescent probe safranine O (λ_ex_ = 520 nm; λ_em_ = 580 nm) with the use of a Fluorat-02-Panorama spectrofluorimeter (Lumex Instruments, St. Petersburg, Russia) [30]. Rat liver mitochondria (0.5 mg/mL) were incubated in the medium containing 210 mM mannitol, 70 mM sucrose, 1 mM KH_2_PO_4_, 10 μM EGTA, 10 µM safranine O and 10 mM Hepes/KOH buffer, pH 7.4. 2.5 mM glutamate *plus* 2.5 mM malate or 5 mM succinate were used as respiratory substrates. In the latter case, the incubation medium was supplemented with 1 μM rotenone.

### 2.9. Production of H_2_O_2_ by Rat Liver Mitochondria

The rate of H_2_O_2_ production by the suspension of rat liver mitochondria was measured with the fluorescent indicator Amplex Red (λ_ex_ = 560 nm; λ_em_ = 590 nm) using Fluorat-02-Panorama spectrofluorimeter (Lumex Instruments, St. Petersburg, Russia) at 37 °C [32]. Rat liver mitochondria (0.15 mg/mL) were incubated in the medium containing 210 mM mannitol, 70 mM sucrose, 1 mM KH_2_PO_4_, 10 μM EGTA, and 10 mM Hepes/KOH buffer, pH 7.4. At the beginning of measurements, horseradish peroxidase (1 a.u./mL) and 10 μM Amplex Red were added to the incubation medium. The amount of the resulting hydrogen peroxide was calculated from the calibration curve. A standard hydrogen peroxide solution was prepared on the day of experiment; its concentration was determined using the molar absorption coefficient E_240_ = 43.6 M^−1^ × cm^−1^.

### 2.10. Statistical Analysis

The data were analyzed using GraphPad Prism 8 and Excel software and were presented as means ± SEM. Statistical differences between the means were determined by a Mann–Whitney U test, where *p* < 0.05 was considered to be statistically significant.

## 3. Results

### 3.1. F16-Betulin Conjugate Induces Death of Rat Thymocytes due to Superoxide Overproduction and Mitochondrial Dysfunction

**Figure 2:**
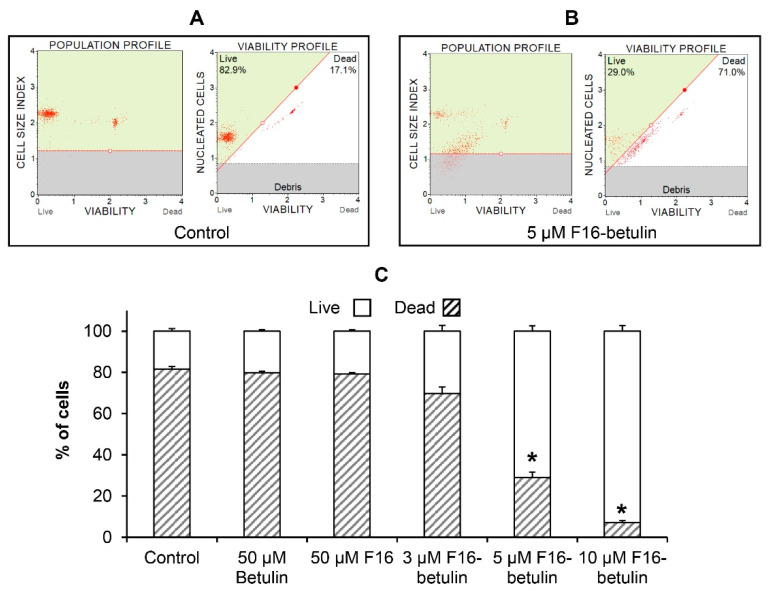
Cytotoxicity of the F16-betulin conjugate against rat thymocytes. Cell viability was assessed using a Muse Cell Analyzer. The upper left quadrant of the viability profile plots (**A**,**B**) represents healthy thymocytes, and the right quadrant shows dead ones. Typical viability profile plots are shown. A similar pattern was observed in four independent experiments. Panel (**C**) demonstrates the ratio (%) of living and dead thymocytes, mean values ± SEM (n = 4) are presented. * *p* < 0.05 (vs. control).

It is known that native betulin, as well as F16, have an antitumor effect [22,33], while their effectiveness is significantly increased by combining into a hybrid conjugate that shows significant cytotoxic effects on certain types of cancer cells [23]. Here, using flow cytometry, we evaluated the *in vitro* cytotoxic effect of the F16-betulin conjugate on healthy rat thymocytes. Figure 2 shows that at a concentration of less than 5 μM the conjugate weakly affects the survival of these cells; however, 5 μM F16-betulin or more causes massive cell death and fragmentation. At the same time, parental betulin and F16 did not have a noticeable effect even at a concentration of 50 μM (Figure 2C, Supplementary Material Figure S1). We previously found that similar concentrations of the F16-betulin conjugate also cause the death of healthy fibroblasts, however, we noted that the conjugate exhibited significantly more potent cytotoxic effects against leukemic cell lines compared to healthy fibroblasts (selectivity index ≥ 10), thus showing a targeted effect against tumor cell lines [23].

**Figure 3:**
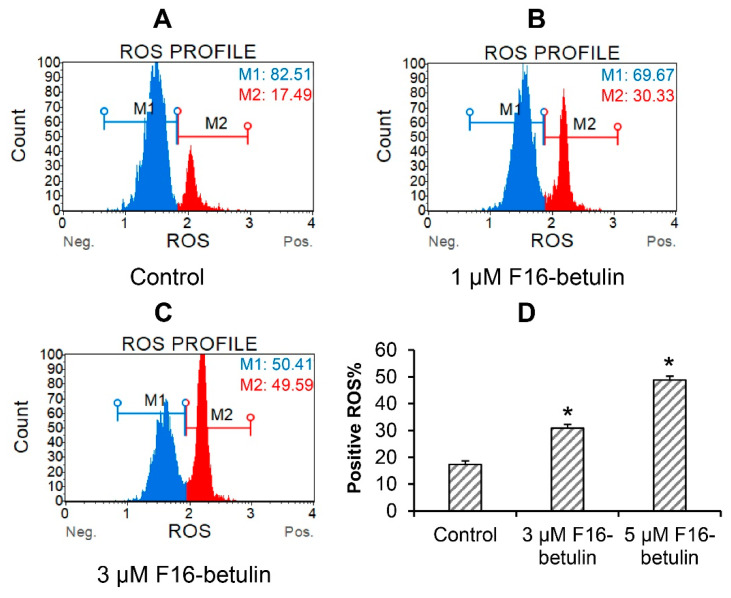
Reactive oxygen species (ROS) burst in thymocytes induced by the F16-betulin conjugate. The ROS profile was assessed using the Muse Oxidative Stress kit (superoxide sensitive). Typical ROS profile plots (**A**–**C**) are shown. A similar pattern was observed in three independent experiments. Panel (**D**) demonstrates the percentage of dihydroethidium-stained (ROS positive) cells (means ± SEM; *n* = 3). * *p* < 0.05 (vs. control).

**Figure 4:**
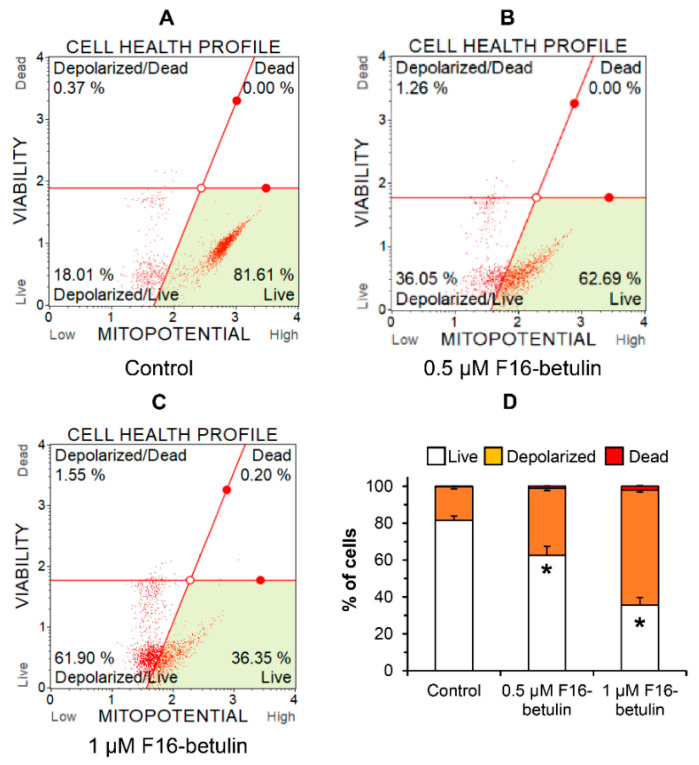
Effect of F16-betulin conjugate on mitochondrial potential of rat thymocytes. The mitochondrial potential of cells was evaluated using the Muse MitoPotential Kit. Typical mitopotential profile plots (**A**–**C**) are shown. A similar pattern was observed in three independent experiments. Panel (**D**) demonstrates the effect of the conjugate on the distribution of cells losing mitochondrial potential (means ± SEM, *n* = 3). * *p* < 0.05 (vs. control).

The antitumor as well as the cytotoxic effect of betulin and other triterpenoids is due to ROS overproduction and the development of oxidative stress in cells [34]. It is believed that mitochondria-targeted compounds based on DLCs have a powerful effect on the redox state of cells, contributing, in particular, to an increase in the generation of ROS in mitochondria [14,15,17]. The same may be expected in the case of the F16-betulin conjugate. Indeed, Figure 3 shows that the conjugate dose-dependently increases superoxide generation in thymocytes, shifting the cell population profile towards dihydroethidium (superoxide indicator)-stained cells. This effect is accompanied by the development of mitochondrial dysfunction in thymocytes. One can see that already 500 nM F16-betulin causes a significant decrease in the mitochondrial potential of these cells (Figure 4B), indicating the suppression of the functioning of organelles. Just 1 μM conjugate causes an almost twofold increase in the depolarizing effect (up to 60% of the total number of cells) (Figure 4C).

### 3.2. F16-Betulin Conjugate Affects the Surface Properties of Mitochondrial Membranes and Induces Their Aggregation

**Figure 5:**
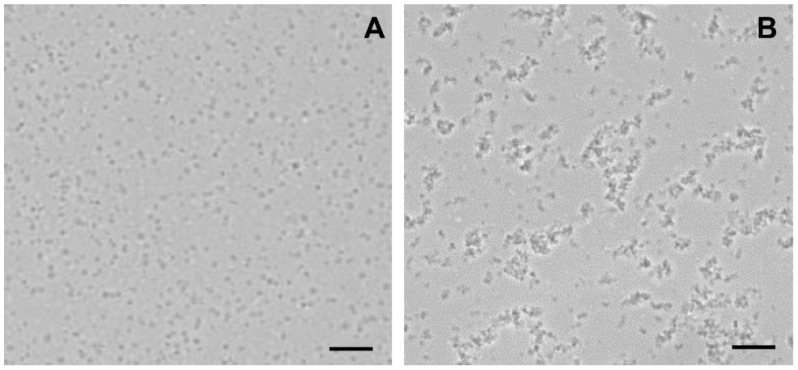
Light microscopy images of rat liver mitochondria incubated with F16-betulin conjugate. Panel (**A**) shows control sample (without additions). Panel (**B**) shows a sample of mitochondria captured after incubation with 30 μM conjugate. Typical images are presented in two sets for each experimental condition. Scale bar is 10 μm.

We have previously demonstrated that betulin exhibits pronounced membranotropic properties, facilitating the aggregation and permeabilization of biological membranes, including mitochondrial and artificial membranes [34]. One could assume that F16-betulin conjugate, possessing similar properties, will accumulate in mitochondrial membranes and affect the surface properties of organelles and their components. To test this, in the next part of the work we used mitochondria isolated from the liver of laboratory rats to evaluate the direct effect of F16-betulin on the behavior of organelles. Using optical microscopy, we studied the state of the mitochondria. In the absence of F16-betulin, mitochondrial particles show a separate location (Figure 5A). However, the addition of the conjugate to the suspension caused the formation of aggregates of mitochondrial particles (Figure 5B).

**Figure 6:**
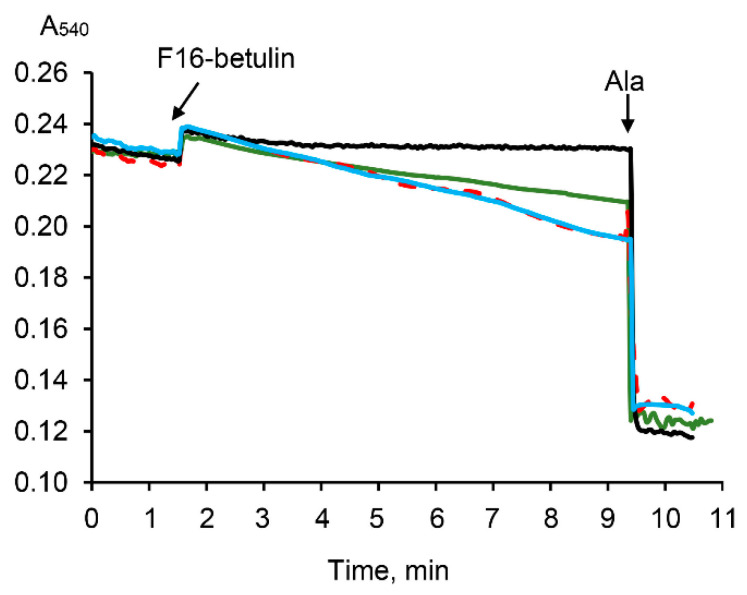
Effect of F16-betulin conjugate on the optical density of a suspension of liver mitochondria. Additions: 30 µM of F16-betulin conjugate in the presence of glutamate *plus* malate (*green line*) and succinate *plus* rotenone (*blue line*). The effect of 1 μM cyclosporin A (CsA) on changes in the optical density of succinate-fueled mitochondria induced by the conjugate is shown by the *red dashed curve*. *Black line* reflects the kinetics of changes in the optical density in the absence of oxidation substrates. Alamethicin (Ala, 5 μg/mL) was used to show the maximum possible decrease in the optical density of the mitochondrial suspension at the end of the recordings. A similar pattern was observed in three independent experiments.

It is known that the aggregation of mitochondria and the permeabilization of their membranes can be recorded by a decrease in the optical density of the organelle suspension [35,36]. Indeed, Figure 6 demonstrates that F16-betulin induces a slow decrease in the optical density of the mitochondrial suspension. This picture is observed only in the presence of oxidation substrates and is most pronounced in the case of respiration of organelles driven by succinate. In addition, this process is insensitive to cyclosporin A (CsA), an inhibitor of the mitochondrial permeability transition (MPT) pore. This may reflect that F16-betulin conjugate induces CsA-insensitive aggregation and swelling of organelles due to permeabilization of the inner membrane.

### 3.3. F16-Betulin Conjugate Induces Mitochondrial Dysfunction

In the next part, we estimated the direct effect of F16-betulin on the functioning of mitochondria and assessed the effect of this agent on the respiration rate of organelles fueled by glutamate *plus* malate (as a substrate of electron transport chain (ETC) complex I) or succinate (as a substrate of ETC complex II) *plus* rotenone. Table 1 and Table 2 demonstrate that 10–20 μM F16-betulin has no effect on oxygen consumption by organelles in states 2 and 3 in the presence of glutamate *plus* malate and succinate *plus* rotenone. However, the addition of F16-betulin leads to a dose-dependent increase in the rate of glutamate/malate-dependent respiration of liver mitochondria in state 4 (Table 1). The maximum effect (20% stimulation of respiration) is achieved at 20 μM concentration of this conjugate. In this case, F16-betulin did not affect the maximal 2,4-dinitrophenol (DNP) -induced respiration of organelles. In addition, F16-betulin conjugate did not affect succinate-fueled oxygen consumption of organelles in both state 4 and state 3U_DNP_ (Table 2). As a result, we noted a 10% suppression of the respiratory control ratio of glutamate/malate-fueled mitochondria induced by 20 μM conjugate (Table 1).

We have noted that the mitochondrial F1F0-ATPase inhibitor oligomycin abolishes the ability of the F16-betulin to increase state 4 respiration rate (Table 1), indicating the ability of the conjugate to disrupt the ADP phosphorylation process. This, in turn, may be due to a decrease in the activity of complexes of the electron transport chain of organelles or reversion of the activity of ATP synthase, leading to ADP conservation and maintenance of a high rate of oxygen consumption in state 4 [37]. We have previously shown that triterpenoids and, in particular, betulin, accumulating in the inner membrane of mitochondria, may significantly affect the redox activity of complexes of the mitochondrial electron transport chain [34]. The same is observed in the case of hybrids of F16 and betulinic acid, which have a powerful modulating effect on the ETC complexes [6]. Examination of the activity of mitochondrial ETC complexes shows that F16-betulin conjugate at a concentration of 20 μM also causes ~40% inhibition of the redox activity of complex I, and suppresses the activity of succinate dehydrogenase (complex II) by 15% (Table 3). One could assume that the pronounced inhibitory effect of F16-betulin conjugate on complex I of the electron transport chain contributes to the suppression of ADP phosphorylation, which manifests itself as an increase in the intensity of oxygen consumption by glutamate/malate-fueled mitochondria in state 4. At the same time, we established a dose-dependent stimulating effect of the conjugate on the redox activity of complex III of the electron transport chain of organelles (20 μM caused ~25% effect). In addition, F16-betulin had no effect on the redox activity of cytochrome c oxidase over the entire range of studied concentrations (Table 3).

**Figure 7:**
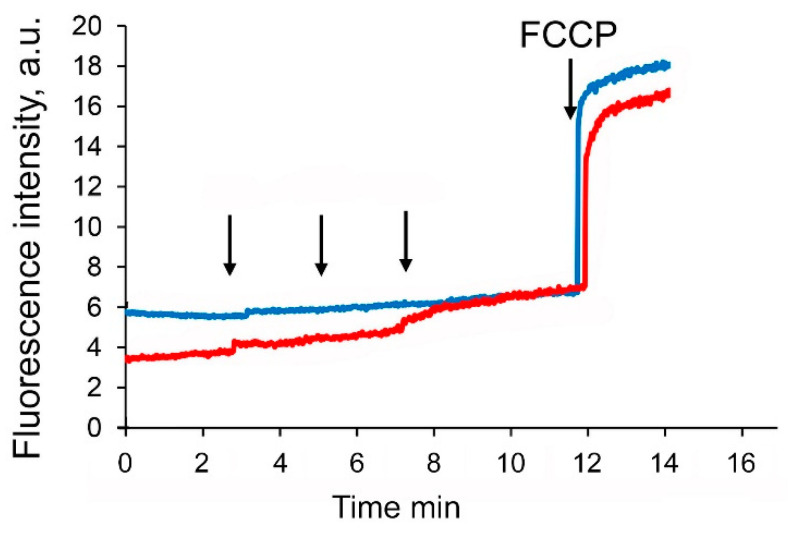
Effects of F16-betulin conjugate on mitochondrial membrane potential. Additions: 5 µM of F16-betulin (shown by an arrow) in the presence of glutamate *plus* malate (*red line*) and succinate *plus* rotenone (*blue line*). 1 μM carbonyl cyanide-p-trifluoromethoxyphenylhydrazone (FCCP) added at the end of the recordings to completely depolarize the inner mitochondrial membrane. A similar pattern was observed in three independent experiments.

It is well known that suppression of complexes of the mitochondrial electron transport chain causes a decrease in the membrane potential of organelles. Indeed, Figure 7 shows that the sequential addition of the F16-betulin conjugate leads to a dose-dependent decrease in the membrane potential of glutamate/malate-fueled liver mitochondria, but at the same time has little effect on the membrane potential of succinate-driven mitochondria.

### 3.4. F16-Betulin Conjugate Induces Overproduction of Hydrogen Peroxide by Mitochondria

Paragraph 3.1 shows that F16-betulin induces ROS overproduction and this may be responsible for the cytotoxic effect of this agent (Figure 3). Mitochondria are considered the main cellular producers of ROS [38]. Here, we evaluated the rate of H_2_O_2_ generation by rat liver mitochondria in the presence of F16-betulin. Table 4 shows that 5–10 μM conjugate causes dose-dependent increase in the rate of H_2_O_2_ generation by glutamate/malate-fueled mitochondria and at the same time has no effect on the generation of hydrogen peroxide by succinate-fueled organelles.

## 4. Discussion

Betulin, like other lupane-based triterpenes, is a common secondary metabolite of many plants. Due to its high hydrophobicity, betulin has a weak biological activity, however, some chemical modifications of its molecule can convert it into a potential therapeutic agent. An effective strategy for increasing the therapeutic efficacy of triterpenes is their conjugation with molecules with targeted properties and, in particular, exhibiting mitochondrial targeting. Indeed, it is known that hybrids of lupane triperenoids (betulin and betulinic acid) with triphenylphosphonium or rhodamine B are able to accumulate in mitochondria and induce mitochondria-mediated cell death and destruction [2,3,4,5,24]. Moreover, these conjugates act more efficiently on various types of tumor cells than on healthy ones, which indicates the selectivity of their effect. Newly synthesized hybrids of betulin and mitochondria-targeted F16 cation also have promising effects on tumor cells [23].

In this work, we found that 5–10 μM F16-betulin shows a powerful cytotoxic effect on healthy thymocytes of laboratory rats, inducing their death and destruction (Figure 2). According to our recent data, F16-betulin also induces the death of healthy cells (fibroblasts), however, we noted that this agent exhibited significantly more potent cytotoxic effects against leukemic cell lines compared to healthy fibroblast cells (selectivity index ≥ 10) [23]. This indicates a targeted effect of F16-betulin against tumor cells. As shown in this work, the cytotoxic effect of F16-betulin conjugate may be due to its effect on mitochondrial functioning. Indeed, one can see that the conjugate-induced death of thymocytes is preceded by a significant dysfunction of mitochondria (Figure 4), which manifests itself in a decrease in the membrane potential of organelles in cells already at 500 nM of this agent. This indicates the targeted transport of the conjugate to mitochondria and disruption of the functioning of the membrane systems of these organelles. In this case, the direct cytotoxic effect of the F16-betulin seems to be due to a dramatic increase in superoxide production by thymocyte mitochondria (Figure 3), causing oxidative stress and related processes.

We used isolated rat liver mitochondria to clarify the direct effect of the F16-betulin conjugate on the mitochondrial apparatus. In this case, we studied the effect of the conjugate on the state of organelle membranes and basic parameters reflecting the functional activity of rat liver mitochondria. We have previously shown that native betulin can easily integrate into the lipid bilayer of the membrane, forming domains enriched in triterpenoid and leading to phase separation [34]. These structural changes presumably contribute to further changes in membrane dynamics, causing loss of their integrity and membrane aggregation/fusion. Here we found that F16-betulin also affects the surface properties of mitochondrial membranes, promoting the aggregation of organelles (Figure 5), leading to sedimentation of mitochondria in suspension, which can be recorded by a decrease in optical density. Moreover, this process was observed only in the case of mitochondrial energization and was insensitive to CsA (MPT pore opening inhibitor). This suggests that F16-betulin is capable of inducing aggregation of organelles by a CsA-insensitive mechanism, which may be accompanied by their permeability.

It is obvious that the modification of the surface properties of mitochondria can lead to a change in the functional activity of mitochondrial transport systems. Indeed, one can see that F16-betulin induces a dose-dependent change in oxygen consumption and oxidative phosphorylation of liver mitochondria. This effect is especially pronounced in NAD-fueled organelles. In this case, we observed an olgomycin-sensitive increase in the rate of oxygen consumption in state 4, indicating a violation of the process of ADP phosphorylation by F16-betulin, apparently due to a decrease in the redox activity of complexes of the electron transport chain or reversion of the complex V (ATP synthase) activity. Indeed, we found that this agent shows a powerful effect on the redox activity of the primary sites of the ETC, significantly inhibiting the activity of complex I, as well as exerting a small inhibitory effect on the redox activity of succinate dehydrogenase (complex II). This, most likely, leads to suppression of ADP phosphorylation and causes a decrease in the respiratory control ratio of organelles, which is most pronounced in the case of NAD-dependent substrates (glutamate *plus* malate). In addition, inhibition of ETC complexes by F16-betulin also destabilized the mitochondrial transmembrane potential—∆ψ (Figure 7), which was also pronounced on NAD-fueled mitochondria and, possibly, due to a powerful decrease in the redox activity of ETC complex I.

It is known that suppression of the redox activity of ETC and its complexes and, in particular, complex I, provokes a powerful ROS burst in mitochondria [38]. For example, it is well known that rotenone, which specifically inhibits the redox activity of ETC complex I, causes a significant increase in ROS generation [39]. One can see that the F16-betulin conjugate significantly inhibiting the activity of complex I, also stimulates the generation of hydrogen peroxide in organelles fueled by glutamate *plus* malate. However, this agent did not affect H_2_O_2_ production in mitochondria oxidizing succinate, the substrate of complex II. It can be assumed that this effect of the F16-betulin conjugate also causes ROS burst in rat thymocytes (Figure 3), leading to massive superoxide generation, contributing to oxidative stress and cell damage.

The data obtained in this work allow to conclude that the powerful cytotoxic impact of F16-betulin may indeed be associated with its noticeable effect on the functioning of membrane electron transport systems that underlie the bioenergetics of organelles. This, apparently, promotes mitochondrial dysfunction and active ROS generation leading to oxidative stress and *in vitro* cell death. The effects of the conjugate are expressed both on healthy cells and on cells of various tumor lines and only further studies will allow us to confirm the selectivity of the F16-betulin conjugate in relation to tumor cells of various lines showing a high mitochondrial potential.

## Figures and Tables

**Table 1:** Effect of F16-betulin on oxygen consumption by rat liver mitochondria fueled by glutamate *plus* malate.

Additions	State 2	State 3	State 4	State 3U_DNP_	RC
	nmol O_2_/min/mg Protein				Relative Units
Control	3.82 ± 0.44	23.56 ± 0.84	4.37 ± 0.13	23.23 ± 2.41	5.40 ± 0.08
F16-betulin conjugate					
10 μM	4.49 ± 0.43	25.80 ± 1.00	4.77 ± 0.15	27.72 ± 0.79	5.41 ± 0.04
20 μM + 1 µg/mL oligo	4.64 ± 0.54	26.32 ± 0.58	5.34 ± 0.18 * 4.21 ± 0.22 #	28.09 ± 1.39	4.93 ± 0.06 *

Oxygen consumption by mitochondria was driven by glutamate *plus* malate. Respiratory control ratio (RC = state 3/state 4). Table shows means ± SEM (*n* = 3). 1 µg/mL oligomycin (oligo) added at state 4 mitochondrial respiration. * *p* < 0.05 (vs. control), # *p* < 0.05 (vs. respiration rate in the absence of oligomycin).

**Table 2:** Effect of F16-betulin on oxygen consumption by rat liver mitochondria fueled by succinate.

Additions	State 2	State 3	State 4	State 3U_DNP_	RC
	nmol O_2_/min/mg Protein				Relative Units
Control	9.86 ± 0.91	44.62 ± 2.30	9.14 ± 0.67	49.54 ± 2.96	4.91 ± 0.15
F16-betulin conjugate					
10 μM	9.56 ± 1.00	44.91 ± 0.55	9.39 ± 1.27	51.16 ± 0.94	4.87 ± 0.44
20 μM	9.85 ± 0.11	40.67 ± 0.83	9.25 ± 0.43	43.25 ± 0.51	4.41 ± 0.12

Oxygen consumption by mitochondria was driven by succinate in the presence of rotenone. Table shows means ± SEM (*n* = 3).

**Table 3:** Effect of F16-betulin on the activity of complexes of the electron transport chain of liver mitochondria (expressed in percentage vs control).

	Complex I	Complex II	Complex III	Complex IV
10 µM conjugate	99.76 ± 3.74	89.51 ± 1.09 *	112.97 ± 3.31 *	103.49 ± 3.02
20 µM conjugate	62.27 ± 1.76 *	85.68 ± 3.04 *	124.66 ± 1.37 *	91.83 ± 3.77

In the absence of conjugate (control), the activities of electron transport chain (ETC) complexes I, II, III and IV were 381 ± 13, 424 ± 16, 688 ± 12 and 482 ± 15 nmol/min/mg protein respectively. Redox activity in the absence of F16-betulin was taken as 100%. Table shows means ± SEM (*n* = 3). * *p* < 0.05 (vs. control).

**Table 4:** Effect of F16-betulin on the rate of H_2_O_2_ generation by mitochondria (H_2_O_2_/min/mg protein).

	Glutamate/Malate	Succinate/Rotenone
Control	146 ± 6	164 ± 7
5 µM conjugate	185 ± 8 *	168 ± 2
10 µM conjugate	205 ± 15 *	164 ± 9

Glutamate *plus* malate or succinate *plus* rotenone were used as respiratory substrates. Table shows means ± SEM (*n* = 4). * *p* < 0.05 (vs. control).

## Data Availability

The data presented in this study are available on request from the corresponding author.

## Supplementary Materials

The following are available online at https://www.mdpi.com/article/10.3390/membranes11050352/s1, Figure S1: Effect of betulin, F16 and F16-betulin conjugate on thymocyte viability. Cell viability was assessed using a Muse Cell Analyzer. The upper left quadrant of the viability profile plots represents healthy cells, and the right quadrant shows dead cells. Typical viability profile plots (A–F) are shown. A similar pattern was observed in three other independent experiments.
