# Supplementary material for: Effect of F16-Betulin Conjugate on Mitochondrial Membranes and Its Role in Cell Death Initiation
Source: Membranes (Basel). 2021 May 10;11(5):352. doi: 10.3390/membranes11050352 (PMC8151401; doi:10.3390/membranes11050352)
Supplement: Supplementary file 1 [file membranes-11-00352-s001.zip › membranes-1202227-supplementary.pdf]

membranes

Supplementary Material: Effect of F16-Betulin Conjugate on Mitochondrial Membranes and Its Role in Cell Death Initiation

Mikhail V. Dubinin, Alena A. Semenova, Darya A. Nedopekina, Eldar V. Davletshin, Anna Yu. Spivak and Konstantin N. Belosludtsev

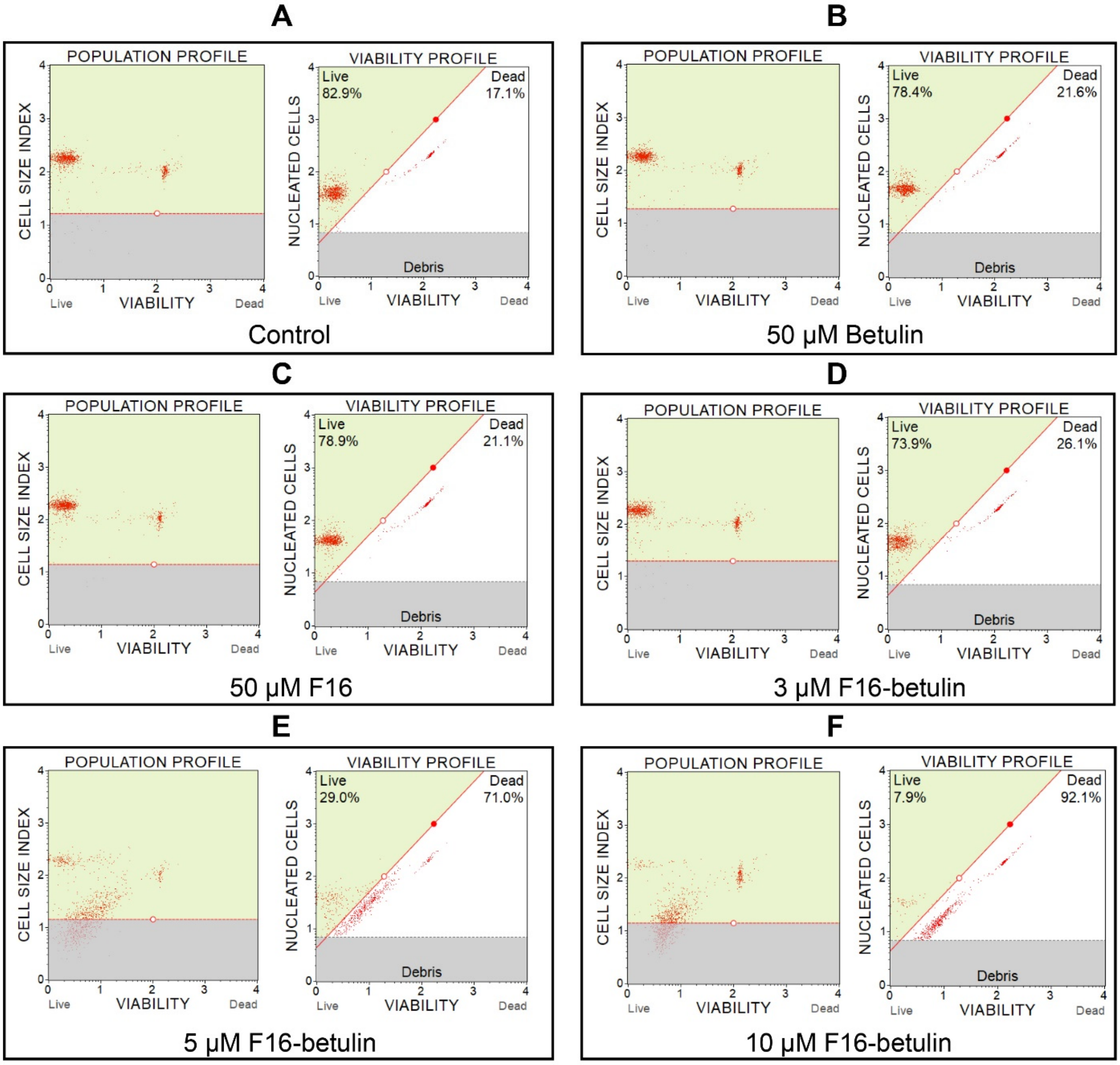

Figure S1. Effect of betulin, F16 and F16-betulin conjugate on thymocyte viability. Cell viability was assessed using a Muse Cell Analyzer. The upper left quadrant of the viability profile plots represents healthy cells, and the right quadrant shows dead cells. Typical viability profile plots (**A**–**F**) are shown. A similar pattern was observed in three other independent experiments.
